# VORTEX: Network-Driven Opportunistic Routing for Ad Hoc Networks

**DOI:** 10.3390/s23062893

**Published:** 2023-03-07

**Authors:** Ryo Yamamoto, Taku Yamazaki, Satoshi Ohzahata

**Affiliations:** 1 Graduate School of Informatics and Engineering, The University of Electro-Communications, Tokyo 182-8585, Japan; 2 College of Systems Engineering and Science, Shibaura Institute of Technology, Tokyo 135-8548, Japan

**Keywords:** ad hoc network, adaptive routing hierarchization, MANET, opportunistic routing

## Abstract

The potential of ad hoc networks, which enable flexible and dynamic network establishment only by mobile terminals equipped with wireless communication devices, has recently attracted attention for the coming IoT era. Although the nature of ad hoc networks shows the advantages of their autonomous and distributed network management, a manifestation of drawbacks owing to the nature of wireless communication and the mobility of terminals are inevitable. Many routing protocols have already been proposed to address the issues by adapting to nature and achieving a certain level of improvement. However, the routing protocols still suffer from difficulties in information collection for routing and adaptive route management during communication. Moreover, there is another issue that end pair-based routing procedures prevent other end pairs from reusing the routing information effectively. To address the drawbacks of conventional routing protocols, this paper proposes VORTEX, a novel routing protocol that employs an opportunistic routing strategy using hierarchization. One of the characteristic features of VORTEX is its network-driven opportunistic forwarding, in which packets travel toward destination terminals using hierarchy as a guide without conventional route discovery procedures. Moreover, another characteristic feature of VORTEX is that the hierarchical structure also contributes to adapting to communication environment changes in an autonomous manner. In other words, VORTEX enables flexible network-wide information-based routing only with the locally collected information. The simulation results show that the proposed VORTEX could provide better service quality and reliability with improved efficiency compared to the conventional routing protocols. Furthermore, the most significant contribution is not only in the communication performance but also VORTEX omits route discovery or route maintenance from routing protocols, and formed networks themselves have a function to deliver packets toward destination terminals.

## 1. Introduction

Recently, wireless multi-hop networks such as ad hoc networks have been considered to be a promising architecture since the autonomous, self-organized, and distributed network management would play an important role in the communications of IoT (Internet of Things) or in situations where infrastructure support is expected such as disaster sites. Such a self-organized nature of ad hoc networks enables us to provide communication opportunities, and this can be realized only with terminals composing the networks. Thus, there are some advantages in terms of deployment cost, environment adaptability, responsiveness, and so forth. Against this background, wireless multi-hop communication is thought to be a promising technology for deploying networks where infrastructure support is not available. However, ad hoc networks generally use wireless communication for data transmission, which causes reliability degradation. Moreover, the mobility of terminals in ad hoc networks results in a change in network topology. In other words, the connectivity of terminals changes momentarily, and this makes it difficult to establish stable end-to-end routes in the networks.

There are many routing protocols in ad hoc networks to address the unique communication environment. The conventional routing protocols for ad hoc networks can be broadly classified into three categories: proactive (table-driven), reactive (on-demand), and hybrid. The proactive routing protocols such as OLSR (optimized link state routing) [1] continuously manage forwarding routes in the advance of the actual forwarding procedure, whereas the reactive routing protocols such as DSR (dynamic source routing) [2] and AODV (ad hoc on-demand distance Vector) [3] establish end-to-end routes after communication demands arise at source terminals. Hybrid routing protocols generally take advantage of these protocols to achieve better routing performance. However, these conventional unicast routing protocols generally have a limitation in routing flexibility, which is mainly derived from the single communication pair-based routing procedure and end pair-based route management.

A novel routing paradigm named opportunistic routing (OR) has been proposed to address the former issues by exploiting the broadcast nature of wireless communication [4,5]. Unlike the conventional routing protocols, OR does not rely on a specific end-to-end route to forward packets from a source terminal to a destination terminal, but selects optimal forwarders on a hop-by-hop or packet-by-packet basis. This forwarding principle also allows it to forward packets with better link quality and results in simultaneous multi-path forwarding, which greatly improves end-to-end reliability.

However, these conventional OR strategies have drawbacks in gathering routing information and adapting to dynamic network environments. This is because the eligible forwarder selection for ORs requires extensive information such as ETX (expected transmission count), and scalability limitations occur as the network size increases. Moreover, a routing protocol with a fixed route or guide performs reasonably well in stable network environments while its performance degrades in dynamic environments due to its rigid routing procedure.

Therefore, the problems that should be addressed in ad hoc network routing protocol are summarized as the problem of adaptation to terminal mobility. For more details, difficulties in the following points should be addressed to overcome the problem.

- Route discovery/Route maintenance;
- Information collection for routing procedure;
- Handle terminal participation and leave.

In addition, conventional routing protocols for ad hoc networks are generally designed for a specific end-to-end paired terminal, which also degrades the efficiency of information gathering, route discovery, and route maintenance.

Against the above-stated backgrounds, this paper proposes VORTEX, a novel routing protocol that uses hierarchical OR, to overcome the above problems from the following perspectives, and also stated as the research objectives.

- Opportunistic forwarding with hierarchical structure;
- Network-driven adaptive packet forwarding;
- Efficient and effective route reuse,

VORTEX employs an opportunistic forwarding strategy to improve reliability aiming to adapt to the dynamic communication environments of ad hoc networks. Moreover, the hierarchically structured networks contribute to managing forwarding routes without initiating conventional route discovery procedures to find an individual guide for opportunistic forwarding. In addition to that, there is the advantage that the structured networks also become guides for other end terminal pairs. In other words, once the structured networks are established, the information can be a guide for every end terminal pair in the networks, namely, network-driven packet forwarding, which does not require end-to-end pair-based routing procedures, can be realized by the structure for better routing and network management flexibility.

The expected contributions derived from VORTEX can be summarized as follows.

- End-to-end reliability improvement derived from multi-path forwarding strategy of OR;
- Prompt forwarding initiation omitting conventional route discovery procedure;
- Reduction of the number of control packets for route management;
- Relieve burdens of end pairs by network-driven forwarding strategy based on hierarchical structures;
- Effective reuse of routing information with the hierarchical structure.

In other words, the OR in VORTEX contributes to improving end-to-end reliability with multi-path forwarding and the hierarchization contributes to effective and efficient routing information management across the network.

The rest of this paper is organized as follows. In Section 2, conventional routing protocols for wireless multihop networks are briefly introduced. Section 3 describes the details of the proposed OR, VORTEX, along with its hierarchization and forwarding procedures. The performance evaluations are conducted in Section 4 and Section 5 concludes this paper.

## 2. Related Works

Many routing protocols have already been proposed to address the unique communication environment of MANET as briefly explained in the introduction. Many researchers continuously propose effective protocols based on the basic MANET routing protocols with more flexible routing procedures to improve routing performance. For example, in addition to the conventional routing protocols, some metaheuristic-based routing approaches have been proposed to optimize routes in networks [6,7]. Moreover, another approach that cloud-integrated routing and forwarding in MANET to provide a further application of the networks [8].

Here, this paper briefly classifies the conventional routing protocols, especially ORs, from the viewpoint of (1) Information collection, (2) Forwarder selection, and (3) Path selection as shown in Table 1. As Table 1 shows, most ORs rely on network-wide information for their route or forwarder selection, whereas the conventional unicast routing and our proposed methods are classified into local information-based. The following paragraphs briefly introduce the operation of the protocols. Note that, since this paper, as well as our proposed method VORTEX, focuses on routing protocols that have generality and are applicable to various types of networks, solution-specific routing is beyond the scope of this paper.

**Local information-based routing protocols**

The protocols classified into this type do not require network-wide information for route or forwarder selection, and only require local information to perform the routing procedure. DSR [2] and AODV [3], the representative conventional unicast routing protocols for MANET, require route discovery procedures when they do not have valid route information to destination terminals. They also take a single path-based forwarding procedure with the discovered route to the destination terminals. The main difference between the protocols is that DSR manages the end-to-end routes, whereas AODV only manages the next or previous forwarder for a designated destination terminal. Some improved routing protocols such as FT-AORP [9], CORA [10], EORB-TP [15], and AOMDV-GA [6] have been proposed aiming to achieve better routing and communication performance. However, the improved protocols are generally designed for a specific field and are not sufficiently applicable to other fields.

CHORUS (clustered hierarchical opportunistic routing using stability information) [13] and CHOR (clustered hierarchical opportunistic routing) [14], our previous works, also use local information to form clusters and discover guides for opportunistic forwarding. Note that CHORUS is the former version of CHOR and this paper only explains CHOR. CHOR first assigns two types of clusters, local cluster (LC) and regional cluster (RC). LC and RC are established based on the neighbor terminal degree information and CHOR assigns hierarchy information to each cluster head (CH) based on the clustering results. After the clustering and hierarchization, MANET would have a virtual gradient where CH of RC represents the top of the gradient. CHOR then starts the route discovery procedure in the same way as the conventional discovery procedure. The unique feature of CHOR in the discovery procedure is that CHOR degenerates the collected route information and uses the degenerated route information as a guide for forwarding. Thus, the information contains only the nearest CH information and some rendezvous relay terminals. That is to say, packets forwarded by CHOR basically climb up the gradient toward the rendezvous relay terminals or the CH, and then descend the gradient towards destination terminals.

One of the advantages of the above-stated routing protocols is the light and simple routing operation. In other words, it is not necessary for them to gather information from entire networks for the routing procedure, and they also have a certain degree of rapid adaptability to changes in the network environment. However, the simple procedure does not always result in the optimal solution in the sight of comprehensive route management. That is to say, the individual route establishment may fail to use network resources or reuse other routing information efficiently.

**Network-wide information-based routing**

Most ORs make use of network-wide information for the forwarder selection or forwarding decision. Several OR protocols have been proposed and the following ExOR and GeRaF are representative legacy ORs.

ExOR [18,19] uses ETX (estimated transmission count), which is calculated from the inverse of the transmission success rate of each link collected from entire networks, to minimize the number of end-to-end forwarding with prioritized forwarders. In ExOR, the highest priority will be given to the terminal with the smallest ETX to forward packets to a destination terminal and ETX-based priority will be assigned to other forwarders in the same manner based on comprehensive ETX information. The forwarding procedure of ExOR is based on the assigned priority and ExOR appends the set of eligible forwarder lists to a packet header to be able to make a forwarding decision on each forwarder between a source and destination terminal.

GeRaF [12] is a location-based OR that selects a terminal closer to a destination terminal as the next forwarder. In GeRaF, terminals determine their geographic location using GPS and calculate the relative geographic relationships between terminals within the entire network prior to actual forwarding. The terminals periodically broadcast HELLO messages to exchange the relative geographic location among the terminals in the network, that is, a source terminal knows the destination terminal location prior to actual forwarding. The source terminal initiates packet forwarding by exchanging RTS and CTS packets which are different from the ones in the MAC layer. In the RTS/CTS exchange, source terminals add their own location and destination terminals to the RTS packet header. The receiver of the RTS packet checks its location and replies CTS packet in response to the received RTS packet if the receiver location is closer to the destination terminal compared to the source terminal. The CTS packet reply is performed sequentially from the terminal closer to the destination terminal to the further terminals. Then, the source terminal forwards the packet to the terminal that is the closest to the destination with higher priority. GeRaF repeats the same procedure recursively until the packet reaches the destination terminal.

For more improvement of routing performance, routing protocols enhancing the legacy ORs have been proposed [22,23,24,25,26,27]. Wide-ranging strategies such as network coding, geographic location-based, and cross-layered approaches, have been taken to address the drawbacks of conventional routing protocols. Although scrupulous routes or topology management could improve performance in specific environments, complex routing procedures are vulnerable to the fluctuations of a MANET communication environment. Therefore, we would explain routing protocols with a tolerance to the fluctuation and layer-independent ones to improve their applicability to various environments.

In [16], the authors proposed a routing protocol using a bloom filter called HRAN (heat routing for ad hoc networks). HRAN uses the concept of “Heat gradients” that indicate the distance to a specific destination terminal, that is, the terminals closer to the destination terminal feel hotter than the terminals further to the destination terminal. The bloom filters in HRAN are used to create a heat map in each terminal which classifies the neighboring terminals around the destination terminal into several heat levels and creates a heat zone according to the level. With the zone assignment, HRAN starts its route discovery procedure and the route request is forwarded along the assigned heat zone, namely heat gradients. Once the request reaches the destination terminal, the destination terminal sends back a route reply using the backward route that had already been created during the request forwarding procedure. Then, the source terminal starts data packet forwarding and HRAN also creates a heat tunnel which assigns a “hotter” zone along the discovered route for further route discovery. lLCAR (least-cost any-path routing) [20] introduces a more flexible routing procedure that treats all possible trajectories from a source terminal to the destination terminal as a union and uses candidate relay terminals to minimize the forwarding costs. LCAR first defines the cost from the viewpoint of unicast links and link reliability. Then, LCAR combines the costs of candidate end-to-end routes to calculate the integrated end-to-end cost. Based on the calculated cost, LCAR selects the best receiver packet-by-packet and forwards the packets to the destination terminals. However, inaccurate cost information causes duplicate and redundant relay selection and degrades forwarding efficiency. Thus, LCAR has the ability to eliminate such a duplication based on the distance to a destination terminal and the cost of duplicate relays.

As another application of OR, TDiCOR [21], SOAR [11], and OxDSR [17] are proposed for WMNs to flexibly adapt to dynamic state changes. TDiCOR introduces the concept of MISO (multiple input single output) into OR and ETX is used for forwarder candidate selection. Moreover, cooperative acknowledgment and cooperative data transmission are also considered. The cooperative acknowledgment is to confirm packet forwarding by a previous sender because the sender does not care about the responsibility of the next forwarding and just cares about its forwarding. Therefore, TDiCOR uses the acknowledgment to hand over the responsibility to the next hop. The cooperative data transmission is to forward packets with multiple routes to gain route diversity. However, on the basis of MISO, only one terminal takes the responsibility of forwarding in its 1-hop neighbor and others wait for further forwarding if the attempt fails.

SOAR introduces ETX-based shortest route selection and priority-based forwarding to minimize duplicated forwarding maintaining the route diversity of OR. SOAR first selects the shortest route towards a destination terminal and the smallest ETX route will be chosen as the route. The forwarding of SOAR is done by a straightforward procedure where a lower ETX terminal to a destination terminal in a forwarder list has a higher forwarding priority. The forwarder list is generated at a source terminal and includes intermediate terminals satisfying the following conditions.

- ETX of the forwarding terminal for the destination terminal is lower than that of the previous hop terminal on the shortest route;
- ETX of the forwarding terminals ETX for the terminal on the shortest route is within a threshold;
- ETX between any pair of the candidate forwarding terminals is within a threshold.

OxDSR also uses the same concept as SOAR which uses an end-to-end route as a guide to forward packets with diverse routes. OxDSR first establishes an end-to-end route using DSR. Then, OxDSR uses EAX (expected any-path transmissions) to prioritize forwarders by calculating OTC (opportunistic transmission count). OxDSR uses the prioritized forwarders as a relay to the second forwarder for packets, that is, OxDSR uses hop-by-hop OR to forward packets to the next forwarder.

## 3. VORTEX: Variable Opportunistic Routing Transferring Endpoint Expense

### 3.1. Overview

As we have explained the strategies and principles of forwarding in ORs, ORs can achieve better routing performance than conventional unicast routing protocols, especially in lossy environments. Although the conventional ORs have achieved superior reliability and efficiency, most of the protocols take end pair-based routing procedures and the overall network resource usage has not been considered. In other words, a union of routing information between end pairs is not effectively reused for other end pairs even though some protocols maintain forwarding histories for further communication. Moreover, the difficulty of gathering routing information from the entire network prevents the protocols from adapting to changes in the communication environment. Therefore, VORTEX, our proposed OR, employs the following routing strategies for improved adaptability to communication environments, load-balanced forwarding, and efficient use of network resources.

- Tier assignment based on a degree of terminal;
- Tier-based prompt forwarding without route discovery;
- Treat network as a union of forwarding information.

The conventional OR protocols generally adopt a “flat” routing procedure in which all the terminals in a network play the same role. However, the flat routing principle might be an obstacle when we consider the overall network management due to the scalability issues derived from the computational and information-sharing complexity. Therefore, VORTEX takes hierarchy into account as we had already proposed the hierarchy-based clustered OR, CHOR, in [14]. The main difference between CHOR and VORTEX can be summarized as only CHOR requires the clustering procedure to establish forwarding guides as explained in the following paragraph.

Another characteristic feature of VORTEX is its prompt forwarding procedure, whereas conventional ORs including CHOR generally require route discovery-like forwarding guide establishment. One of the advantages of omitting the discovery is its improved adaptability to changes in the communication environments because the conventional ORs require the guide for forwarding eligibility confirmation and they should alter the guide according to the changes. On the other hand, the hierarchical structure and opportunistic forwarding strategy of VORTEX enable adaptive forwarding without the use of such guides if the hierarchical structure is properly maintained. Thus, the hierarchical structure becomes a comprehensive guide for multiple end pairs and VORTEX does not require an individual guide for each end pair. Eventually, the hierarchical guide establishment by VORTEX creates backbone-like network structures that could make opportunistic forwarding efficient. This characteristic is achieved by enforcing reasonable restrictions on forwarding direction and the backbone-like structure guides packets in the appropriate direction toward destination terminals. In other words, the hierarchical structures of VORTEX become help packets to travel appropriate routes in a similar way to reactive P2P overlay routing.

In addition to the above, VORTEX adopts two different control phases, namely network initialization, and network management. In general situations, the network initialization phase is invoked only when the first communication occurs or terminals intentionally form a network for further communication demands. Thus, VORTEX usually operates in the network management phase once the initialization is completed. In the network management phase, VORTEX performs three procedures according to the required process, namely participation, leave, and update procedures. These procedures are independently invoked as needed to deal with topological changes in the network.

### 3.2. Network Initialization

**Figure 1:**
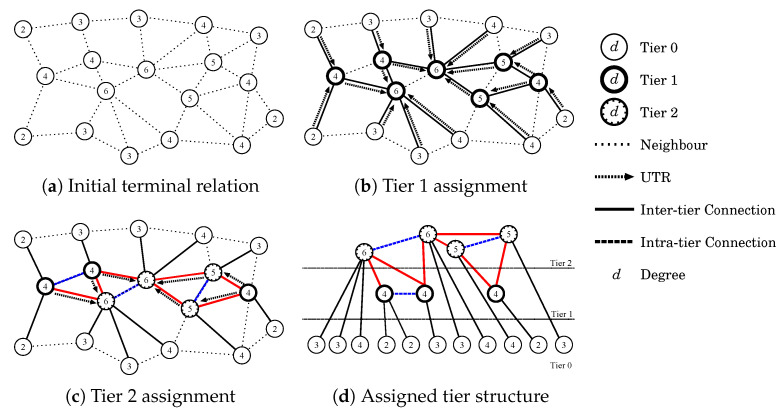
Tier assignment in VORTEX.

As we explained in Section 3.1, the proposed method, VORTEX, takes into account the hierarchical structure to form networks. Although VORTEX uses a similar strategy to CHOR, VORTEX omits the clustering procedure and only adopts the concept of CHOR. This is because even though clustering is an effective strategy for ad hoc networks, cluster-based route discovery is still required to create a forwarding guide and it degrades the routing adaptability to a certain degree. Moreover, the costs for cluster maintenance and participation/leave handling increase according to the number of terminals. The detailed initialization procedure will be described in the following with Figure 1.

VORTEX invokes the initialization procedure when it begins to form a network for the first time. There are several events that can be a trigger for the initialization procedure such as the receipt of a HELLO message, communication request, and so forth. If the initial network size is small and terminals participate after the initialization procedure, the initialization procedure is firstly called afterward the participation procedure explained in Section 3.3 is called. In this subsection, we will explain the initialization procedure by plain descriptions with a relatively large network to make it easy to understand. The initialization procedure in VORTEX first assigns tiers (in this paper, we defined tier 0 to be the lowest tier for the further extension of tier levels) to the component terminals of the network. In this paper, we assume that each terminal periodically broadcasts a HELLO message among its neighbors to notify its existence. Thus, each terminal can perceive how many terminals exist in the neighborhood by counting the number of unique terminal IDs contained in the messages, namely the degree. VORTEX continuously performs this operation to determine the neighbor relationship for further network management. The components of the HELLO message are shown in Table 2 and all the values except for Terminal ID are initialized to a negative value at the beginning.

After a certain number of HELLO messages have been exchanged, VORTEX shifts to the tier assignment phase similar to the clustering procedure of CHOR. The threshold value for the shift is basically set to three exchanges since at least two exchanges are required to observe their own information and another exchange is required to suppress timing-related issues that generally occur in unsynchronized systems. In this phase, each terminal checks the degree of the terminal and its neighboring terminals as shown in Figure 1a. The terminal then sends UTR (upper tier request) to the neighbor terminal that has the largest degree among the neighbor terminals, if the largest one is not itself. In the case of the largest one is the terminal itself, the terminal autonomously set its tier level to 1. Otherwise, the receiver terminal of UTR sets the tier level to 1 as shown in Figure 1b. Once the tier 1 terminals are set, the terminals subsequently send UTR in the same manner as tier 1 selection to find tier 2 terminals. The receiver of the second UTR then sets its tier lever 2 as shown in Figure 1c. Tier 0 terminals, which are the lowest tier terminals, can simultaneously overhear the UTR during the UTR sending for the selection of tier 2 terminals, and then the terminals can regard the tier level as 0. Through this procedure, VORTEX assigns tiers to terminals as shown in Figure 1d. Note that, the terminals in tier 1 or 2 collect the information from the terminals in the lower tiers using this procedure. The main difference between VORTEX and CHOR in the initialization procedure is that the procedure in VORTEX aims to build a structure for self-guided forwarding without a route discovery procedure, whereas the procedure in CHOR aims to create clusters and elect cluster heads that will be rendezvous points through which packets should travel.

Through the tier assignment procedure, VORTEX assigns higher tiers to terminals that are capable of being a hub for opportunistic forwarding and assigns lower tiers to terminals as leaves of the hierarchical structure tree. Thus, networks would be hierarchically organized according to the eligibility as forwarders and logically central terminals with higher tier levels would establish backbone routes for the entire networks.

### 3.3. Network Management

This section explains how VORTEX handles the participation or leave of terminals as the topology changes due to terminal mobility.

**Participation procedure:** The participation procedure of VORTEX is done with almost the same procedure as its initializing procedure, as described in the following. Note that, the newly participated terminal can recognize itself as a newcomer by examining the tier level. Thus, if the recorded value is negative, the terminal does not belong to any network at the moment.

1. The newly participated terminal first exchanges the HELLO message with its neighboring terminals.
2. After obtaining the information of the degree neighboring terminals, the terminal sends UTR to the terminals in the same manner as the initialization.
3. In this procedure, it is assumed that the newly participated terminal first belongs to tier 0 even though the terminal has a larger degree than the neighboring terminals.
4. The receiver terminal of the UTR sends back the reply to the terminal.
5. The terminal then belongs to tier 0 upon receipt of the reply.

As described above, VORTEX treats the newly participating terminal as a tier 0 terminal because the overall optimization should be performed globally in the update procedure of VORTEX.

**Leave procedure:** VORTEX handles the leave of terminals according to the tier level of the terminals. If the terminal belongs to tier 0, VORTEX does nothing and waits for the update procedure. If the tier level is 1 or 2, VORTEX takes the following procedure. Note that, the leave is autonomously detected by the absence of a HELLO message. In this paper, we assume the threshold to be two consecutive absences of HELLO messages from neighboring terminals and set a tier level to a negative value after a terminal detects the leave.

1. If the leaving terminal belongs to tier 1, the impact of the absence will mainly reach the neighboring terminals in tier 0, and thus the terminals will take an action.
2. The terminals detect the absence of the adjacent tier 1 terminal and then send T-UTr (temporary UTR) to the terminal with the second largest degree terminal.
3. The terminal receiving T-UTR temporarily set its tier level to 1 and notifies it with the HELLO message.
4. If the leaving terminal belongs to tier 2, the impact of the absence reaches not only the adjacent terminals but also the 2-hop adjacent terminals. Therefore, VORTEX tries to minimize the impact by reassigning the tier structure.
5. If there is a tier 2 or tier 1 terminal as the adjacent terminal of the terminal that detects the absence, the terminal sends T-UTR to the adjacent terminal.
6. Otherwise, the terminal does the same procedure in 2 and 3.

**Update procedure:** The update procedure in VORTEX will be performed in a similar manner as the initialization. However, VORTEX performs the update procedure every *i* HELLO message interval. Note that, the beginning of an update procedure will trigger updates of other terminals by overhearing or receiving control messages because VORTEX takes a synchronization-free policy and assumes that the update interval has been previously defined among all the terminals.

VORTEX begins the update procedure by querying its neighbors for their degree and tier level. Then, VORTEX performs the tier reassignment in the following manner.

- If a neighboring terminal with a larger degree is in the higher tier, the terminal will remain silent because VORTEX regards the current tier assignment as locally appropriate.
- If the tier level of the larger degree terminal is the same as the one of the reference terminal or lower than that, the terminal sends UTR towards the larger degree terminal and steps down its tier level.
- The receiver of UTR increases its tier level by 1.
- If the degree of the terminal is larger than that of all neighboring terminals, the terminal shifts its tier level to the higher one, unless the current tier is 2.

### 3.4. Opportunistic Forwarding

The forwarding of VORTEX is based on OR and employs the self-guided forwarding principle for network-driven forwarding. Unlike CHOR which requires the conventional route discovery procedure to collect valid routing information, VORTEX uses the information stored in each terminal that is obtained during the initialization and employs a novel forwarding procedure to opportunistically find destination terminals. The forwarding procedure of VORTEX has two states: Destination location unknown and Destination location fixed. The detailed procedure is described in the following.

**Destination unknown state:** In the destination unknown state, VORTEX forwards packets according to the following rules.

- A source terminal broadcasts a packet to its neighboring terminals.
- The neighboring terminals rebroadcast the received packet if the terminals know the destination direction or if there is the same tier-level terminal that is larger than tier 1.
- The terminals in tier 2 rebroadcast the packet until TTL (time to live) expires.
- Tier 1 terminals that received the packet once rebroadcast the packet to search for the destination terminal in a 2-hop neighbor.

**Figure 2:**
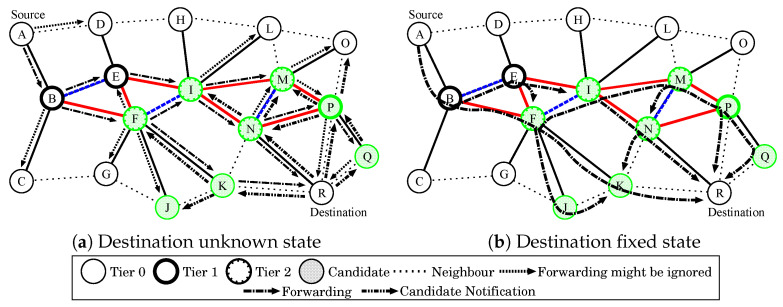
Forwarding in VORTEX.

By repeating the forwarding procedure with the rules, the packet is finally delivered to the desired destination terminal as shown in Figure 2a. However, there is a lot of excess forwarding in this state due to the flooding-like propagation. Then, VORTEX restricts forwarding by using eligible terminals as candidates for opportunistic forwarding. The candidate selection is initiated by the destination terminal using candidate notification messages, and the message is propagated to 2-hop neighbors. Then, the receiver terminal of the message set itself as a candidate forwarder to the destination, if the terminal belongs to tier 1 or tier 2, or is a neighbor of tier 2 terminals. Afterward, the candidate terminals shift to the Destination fixed state.

**Destination fixed state:** In the destination fixed state, VORTEX controls the forwarding as follows.

1. A source terminal broadcasts a packet to its neighboring terminals and VORTEX performs the procedure of destination unknown state.
2. Once the forwarded packets encounter the candidate terminal, the packets are forwarded only among the terminals until they reach the terminals that know the destination terminal as a 2-hop or 1-hop neighbor.
3. 2-hop or 1-hop neighbor terminals set the flag in the packet that represents no more broadcast is necessary for the packet. Then, the receiver terminals of the flagged packet abort further broadcast if they are not a 1-hop neighbor of the destination terminal.
4. The neighboring terminals broadcast the flagged packet to the destination terminals.
5. All the terminals that overhear forwarded packets probabilistically rebroadcast them with the predefined probability *p* to improve route diversity.

By performing the above procedure, VORTEX can opportunistically deliver packets from a source terminal to the destination terminal. That is, VORTEX provides a network as a black box that terminals just forward packets into the network, and it autonomously delivers the packets to the desired destination terminal without any effort on the source terminal. Moreover, the OR-based forwarding of VORTEX can use networks as if terminals composing the networks establish backbone-like topologies since there are simultaneous routes for forwarding and multiple routes for diversion.

In addition to the above procedure, VORTEX uses the candidate information for further forwarding by other terminals. As described previously, the candidate assignment of VORTEX is driven by destination terminals. In other words, the neighboring terminals of the destination terminals become the candidate terminals. Thus, packets destined for the same terminal can be forwarded using the candidate terminals once the packets are delivered to the same destination terminal.

## 4. Performance Evaluation

### 4.1. Simulation Setups

In this paper, we evaluate the performance of VORTEX by comparing it to other routing protocols using simulations. The comparison is made with AODV [3], ExOR [18,19], HRAN [16], LCAR [20], SOAR [11], OxDSR [17], and CHOR [14]. Here, AODV and ExOR are chosen as benchmark protocols since they are the fundamental protocols in ad hoc networks, and HRAN, LCAR, SOAR, OxDSR, and CHOR are chosen since they use a strategy similar to VORTEX that creates a guide for packet forwarding. The simulation setups are described in the following. Note that we use UDP traffic and IEEE 802.11b as the wireless medium to evaluate mainly routing performance since TCP’s performance in MANET is strongly affected by its vulnerable wireless communication nature and its recovery mechanisms blur the pure routing performance. Thus, we avoid the use of rich protocols in other layers to focus on the routing performance.

**Common environment:** We use QualNet [28] 6.1 as the network simulator. In the simulations, terminals are randomly placed in 1000 m × 1000 m square simulation areas and each terminal uses IEEE 802.11b and disabled RTS/CTS mechanism for broadcast-based ORs. The transmission rate is set to 11 Mbps, and the radio range is approximately set to a radius of 100 m. Note that, radio signals attenuate as the distance between the source and receiver increases, and the former radius indicates the threshold at which terminals can correctly receive the packet. Source and destination terminal pairs are randomly chosen from the entire network and bidirectional UDP traffic for 1000 packets of 1024 kbyte is forwarded between the pair terminals in a single forwarding attempt. The traffic generation rate is set to two pairs per second for the duration of the simulations, namely 1000 s. The mobility is defined by RWP (random waypoint) strategy with the pause time set to 0 s. In addition, we use the default values defined in AODV for the HELLO message broadcast interval, route timeout, and allowed HELLO message loss. In other words, HELLO messages are broadcasted every 1 s, and the update interval *i* of VORTEX is set to i=5. The rebroadcast probability *p* in VORTEX is set to p=0.5 in all simulations. In this performance evaluation, we conducted the following three simulations. **Simulation 1** and **2** are conducted to evaluate general routing performance varying terminal density and mobility, and **Simulation 3** evaluates the routing performance varying an information distribution.

**Simulation 1:** In **Simulation 1**, we evaluate the performance of each protocol in terms of terminal density. In this simulation, the number of terminals in the area is changed from 100 to 1000 terminals every 100 steps. The moving speed of the terminals is randomly chosen from 0 m/s to 5 m/s.

**Simulation 2:** In **Simulation 2**, we evaluate the performance from the viewpoint of topology dynamics. In this simulation, 1000 terminals are randomly placed in the simulation area and the moving speed of terminals is fixed and set to 0 m/s to 10 m/s at every 1 m/s step.

**Simulation 3:** In **Simulation 3**, we adopt an information distribution scenario to evaluate the routing performance in a specific application environment. In this simulation, 1000 terminals are randomly placed in the simulation area and the moving speed of the terminal is randomly chosen from 0 m/s to 2 m/s to simulate ambulation. The distribution source is randomly chosen from the whole terminal and the ratio of the source to the entire terminal is changed from 1% to 10%, that is, from 10 terminals to 100 terminals.

Through these simulations, we evaluate the routing protocols from the following aspects: (a) packet delivery ratio, (b) average end-to-end delay, (c) average hop count, and (d) energy consumption. The packet delivery ratio represents the reliability of the routing protocols and is calculated as a percentage of the successful packet delivery to the total packet forwarding. The end-to-end delay and hop count represent the length of the route in terms of time and logical distance, respectively. In general, a shorter hop count results in a shorter delay. However, ORs use appropriate routes opportunistically, and simultaneous forwarding with multiple routes may not use the shortest route. Thus, there might be another relationship between the delay and hop count in ORs, and this evaluation tries to reveal it. The energy consumption evaluates the overall transmission efficiency since ORs generally use multiple and non-shortest routes, which may require more transmission even though they improve end-to-end reliability. In addition to that, unicast routing protocols such as AODV adopt the ARQ mechanism in the MAC layer to improve link reliability, whereas it increases energy consumption. Thus, evaluating the energy required to send a single packet from source to destination represents reasonable overall routing efficiency including reliability.

### 4.2. Simulation Results

**Figure 3:**
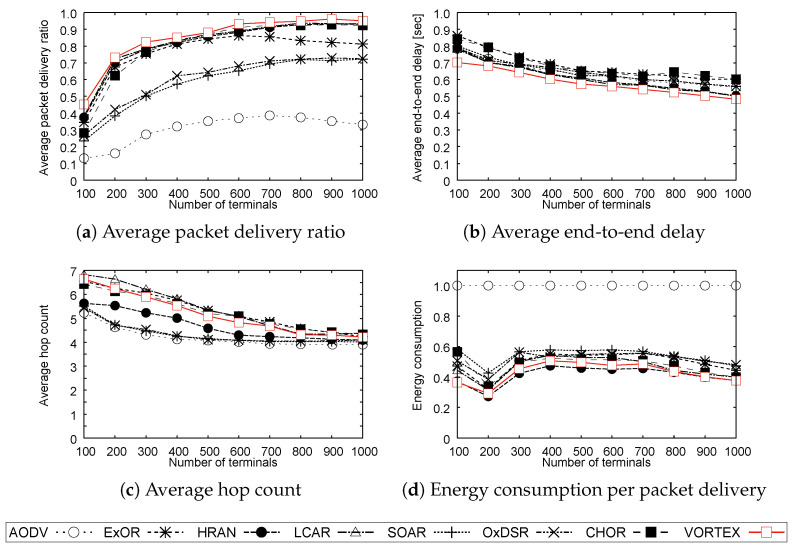
Results of Simulation 1.

**Figure 4:**
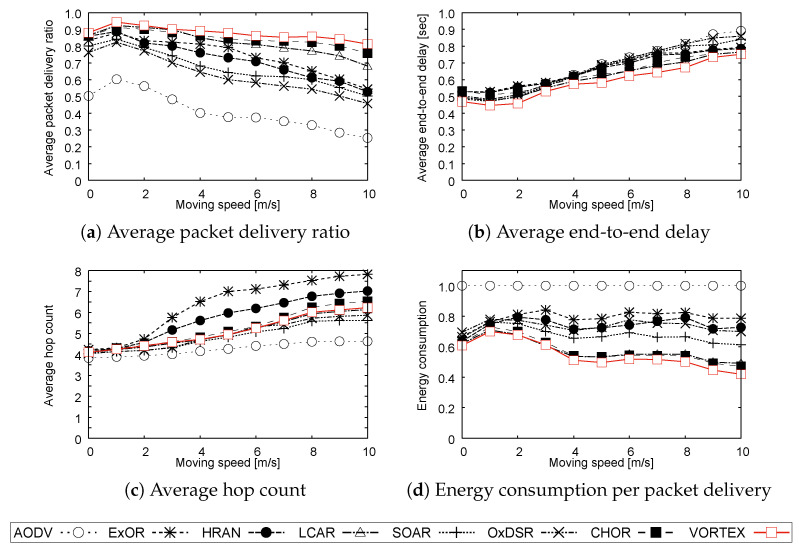
Results of Simulation 2.

**Figure 5:**
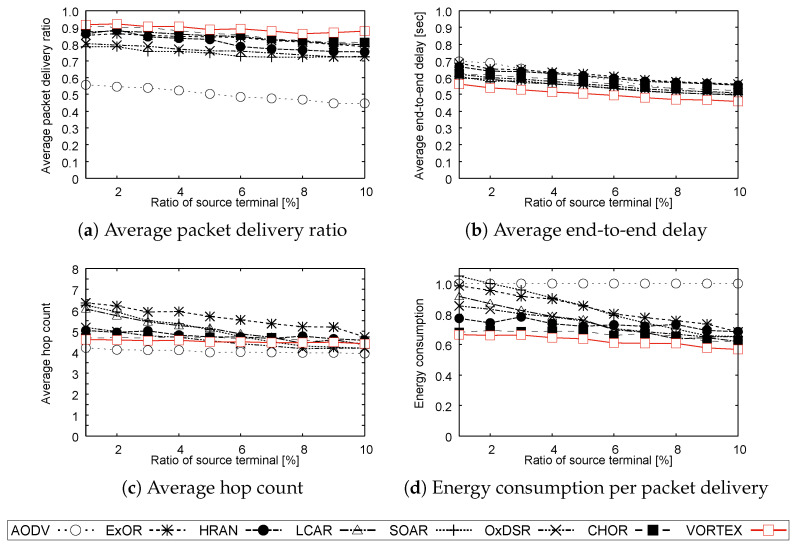
Results of Simulation 3.

Figure 3, Figure 4 and Figure 5 show the results of **Simulation 1**, **Simulation 2**, and **Simulation 3**, respectively. Note that the results of energy consumption per packet delivery are normalized to AODV.

**Simulation 1:**

As shown in Figure 3a, VORTEX could achieve a higher probability of packet delivery compared with conventional unicast routing and ORs. Moreover, the ORs that use the shortest route as a guide, SOAR, and OxDSR, could not improve their reliability, whereas the other ORs can achieve higher reliability. This is because the shortest route does not always result in the best route and may be less reliable than detour routes. Moreover, the shortest route as a guide enforces a certain degree of restriction on opportunistic forwarding and that may degrade the route diversity of OR. On the other hand, ExOR, HRAN, LCAR, CHOR, and VORTEX can achieve higher delivery ratios even in sparse environments. This is due to the fact that their routing procedure can improve route diversity compared to the other routing protocols in this simulation. Moreover, these routing protocols have a better probability when the number of neighboring terminals is larger because they can reap the benefit of route diversity increment to forward packets. However, the result shows that the delivery ratio of ExOR decreases slightly when the number of terminals is greater than 600. This can be explained by its complex routing procedure, that is, the complexity of the ETX calculation and scalability limitations due to the fact that ExOR has to obtain the information from all the terminals in a network. Although some ORs and VORTEX have almost the same characteristics in this result, VORTEX has a slightly higher delivery ratio because VORTEX can achieve more route diversity than other ORs, especially in the destination unknown state. Moreover, the forwarding of VORTEX especially in the state has more routing flexibility than CHOR since packets in CHOR are designed to travel through terminals that are treated as rendezvous points in the degenerated routing table. In other words, packets in VORTEX travel through higher-tier terminals, and more terminals are involved in forwarding. This allows VORTEX to increase route diversity, and this increase contributes to improving reliability.

Figure 3b shows the end-to-end delay which represents the time gap between the initiation time of packet forwarding at a source terminal and the time when the destination terminal receives the packet. The result shows that there is not a large difference between the protocols. Packets forwarded by the shortest route generally have a shorter delay on wired networks. However, in ad hoc networks, the route with the smallest hop count does not always have the shortest delay because communication environment issues such as link quality affect the delay and may result in degradation. Thus, the delay becomes almost the same because ORs can forward packets using appropriate and multiple routes that have a better environment even though the hop count of ORs shown in Figure 3c is larger than the shortest route-based protocols. Moreover, VORTEX can achieve greater route diversity than CHOR because cluster-free hierarchization improves the route selection applicability and this increases the possibility of using the shortest route in terms of delay. This can also be seen from the result of the hop count that VORTEX has a slightly smaller count than ExOR, LCAR, and CHOR.

Figure 3d shows that the ORs could achieve lower energy consumption compared with AODV. This is because that AODV has lower end-to-end reliability as well as lower link reliability than the protocols and requires more retransmission to forward packets to the next hop terminal. Moreover, broadcast-based forwarding also contributes to suppressing the energy consumption per packet forwarding since it can simultaneously forward the same packet to multiple receivers with a single transmission. Figure 3d also shows that there are similar results in energy consumption, which can be explained by the results of packet delivery ratio and hop count. In other words, the protocols with higher delivery ratios have slightly longer hop counts and the protocols with lower ratios have shorter hop counts. Therefore, the comprehensive energy consumption becomes almost the same value since they require an equivalent number of transmissions.

**Simulation 2:**

Figure 4a shows that LCAR, CHOR, and VORTEX can achieve better performance than the other protocols, whereas the others degrade their performance as the moving speed increases. The reason for the degradation in the conventional protocol is mainly due to the difficulty in the route establishment in the high mobility environment. Therefore, the route-based protocols including the tunnel-based protocol, AODV, SOAR, HRAN, and OxDSR, may not be able to establish appropriate routes or guides for forwarding. In addition, ExOR faces the difficulty of collecting valid information in highly mobile environments, which degrades the accuracy of the information and can make it difficult to forward packets with appropriate routes. On the other hand, forwarding in LCAR, CHOR, and VORTEX can dynamically adapt to the topology changes due to terminal mobility with their flexible forwarding strategy. Moreover, the hierarchical forwarding of VORTEX, namely the forwarding that packets climb up and descend the tier gradients, contributes to improving reliability by using multiple routes simultaneously since VORTEX does not require a route-like forwarding guide.

The end-to-end delay and hop count shown in Figure 4b,c represent a similar characteristic in **Simulation 1**. The results show an increasing trend with increasing moving speed due to the difficulty of optimal route selection. However, the opportunistic forwarding strategy in ORs can effectively suppress the delay even though the hop counts increase slightly for the same reason previously explained in the result of **Simulation 1**. In addition to that, we can see that ExOR has a larger hop count than the other protocols. This is because the forwarder list selection in highly mobile environments becomes difficult as explained, and the list becomes obsolete in a short time. This could result in an inappropriate forwarder list with a larger hop count being used in the environment.

Figure 4d shows the same characteristics as with the result of **Simulation 1** that ORs can achieve better energy efficiency than AODV. This fact is derived from the same reason as explained in **Simulation 1** part, that is, ORs could achieve a higher delivery ratio and require fewer retransmissions to recover reliability. In addition to that, the reliability degradation of ExOR, HRAN, SOAR, and OxDSR in high mobility environments also degrades their efficiency, whereas LCAR, CHOR, and VORTEX could achieve almost the same efficiency as in the former simulation. This is because their reliability is relatively higher than the others, especially with CHOR and VORTEX. Moreover, VORTEX achieves higher reliability and a smaller hop count than CHOR, resulting in the highest efficiency. The efficiency improvement of VORTEX is also derived from its forwarding strategy, that is, its hierarchical forwarding strategy both in the destination unknown and fixed states can effectively suppress unnecessary forwarding according to the assigned structure. This is because forwarded packets are routed in logically appropriate directions in the unknown state, and in both logically and physically appropriate directions in the fixed state.

**Simulation 3:**

Figure 5a shows that ORs could achieve better reliability than AODV. This is mainly due to the flexibility of forwarding in ORs, that is, improved route diversity of ORs effectively distributes the concentrated traffic around source terminals, which generally causes reliability degradation. Moreover, every protocol slightly degrades the reliability as the source terminal ratio increases because the concentration can occur at multiple points simultaneously, increasing the number of degradation points throughout the networks. In addition to that, the reliability improvement of VORTEX over other protocols can be explained by its forwarding flexibility while the conventional protocols suffer from the over-concentration of routes and traffic in the area of source terminals and the neighboring area of guide routes for forwarding.

Figure 5b,c show a similar order of performance order as the former simulations. Figure 5b shows the delay gradually decreases as the number of source terminals increases. This is mainly due to the dispersion of traffic and a decrease in queuing delay, which is caused by the increase in forwarding routes. The improvement in VORTEX can be explained by the same reason described in the last paragraph that more flexible route selection in VORTEX adaptively distributes concentrated traffic around source terminals better than the others. Figure 5c shows that the hop count of AODV and VORTEX is almost constant, whereas the others increase their hop count as the traffic concentrates. The increase in the number of hop counts is mainly due to the fact that congested links generally have lower reliability and efficiency, that is, the protocols have a tendency to forward packets detouring such links.

Figure 5d shows characteristic trends compared with the other simulation results that some protocols greatly degrade the energetic efficiency, especially in congested environments. The degradation was mainly due to the decrease in reliability and the increase in hop count. On the other hand, VORTEX could achieve the best efficiency among the protocols because it achieved the best reliability and reasonable hop count increment. In addition to that, the flexible and efficient forwarding strategy of VORTEX contributes to suppressing the number of forwarding, namely the energy consumption, as is the case with the other simulations.

**Discussions:**

Through the above simulations, we have confirmed that the proposed routing protocol, VORTEX, can achieve better performance than any other conventional comparable protocols from the following viewpoints. **(1)** VORTEX could achieve the best reliability among the protocols owing to its diverse route usage suppressing unnecessary forwarding. **(2)** VORTEX could restrain the increase in end-to-end delay owing to its prompt forwarding process and appropriate forwarding route selection with OR even though a certain degree of hop count increase could be observed. **(3)** VORTEX could achieve the lowest energy consumption to deliver a single packet to the destination terminals since it has high reliability and reasonable hop count that requires less packet forwarding compared with the other conventional protocols. In other words, the related results showed that VORTEX achieved (a) 3.92–10.20% improvement in average packet delivery ratio, (b) 7.42–14.89% reduction in average end-to-end delay, (c) 1.79–2.68% reduction in average hop count, and (d) 5.56–9.48% reduction average energy consumption per packet delivery on average. Moreover, CHOR, our previous work, requires a route discovery procedure in a similar way as the conventional routing protocols do to find terminals that would be assigned as rendezvous points through which packets should travel. VORTEX, on the other hand, uses tiered structures as a comprehensive guide for packets to travel and the tier gradient spontaneously directs packets to designated destination terminals suppressing forwarding that go off the appropriate directions. Thus, VORTEX achieved higher adaptability to network state changes and this improves both reliability and efficiency compared with CHOR since the routing performance of CHOR strongly depends on the initial route discovery and CHOR is difficult to change routing tables adaptively during a session. Therefore, we conclude that VORTEX has met the research objectives stated in Section 1 from the following perspectives.

- As for route discovery/route maintenance, VORTEX omits any of them for packet forwarding by using the network itself as a guide for packet forwarding.
- As for information gathering for the routing procedure, VORTEX only uses topological information that can be gathered from neighboring terminals for packet forwarding.
- As with the handling terminal participation and leave, VORTEX handles terminal participation and leaves so as not to impact the existing hierarchy for the first time and a periodical hierarchy update procedure re-arranges the hierarchy according to the topological characteristics of the participated/leave terminals.

We also conclude that VORTEX has acceptable reliability and efficiency in various environments. In other words, VORTEX has a flexible adaptation ability to various environments maintaining its routing performance, especially reliability and efficiency.

However, VORTEX still has a limitation in adapting to topology changes especially when the terminal moving speed becomes higher same as the conventional routing protocols. Therefore, every performance metric degrades as the moving speed increases. Although more stable guide selection in such environments is required to overcome the issue as described in [29], it is beyond the scope of this paper and remains a future study.

For the future development of these research achievements, we are now researching ORs in heterogeneous environments of IoT (Internet of Things) [30]. This is because that heterogeneous environments generally consist of various kinds of communication media, and it requires a rendezvous point for mutual communication between the media. However, the communication environments change from time to time, and difficult to specify the optimal end-to-end route with conventional routing protocols. In addition to that, the heterogeneity also affects the symmetry of the communication range of terminals and the link bandwidth, namely, an increase in unidirectional communication is inevitable, which greatly affects the routing performance. Therefore, handling the asymmetry is an important issue in OR for heterogeneous networks and we have proposed a flag-based symmetry confirmation-based OR [31].

Another use-case for ORs can be found in the field of WMNs (wireless mesh networks), and some studies introduce network coding to WMNs using ORs [32]. This is a similar application of ORs in underwater wireless sensor networks [33,34], and both applications are designed to collect packets in a single location. Thus, an important issue for ORs in the fields is path diversity and it is not required to address some issues that VORTEX has done in this paper. However, network-driven forwarding in VORTEX can also be applied to the fields and may have sufficient performance if the strategy of VORTEX is modified to be able to handle their communication characteristics.

Moreover, we are now researching an OR that can handle link bandwidth asymmetry to maximize available network traffic. By combining the achievements of the research, we believe that communication in the IoT can become more flexible and efficient. Furthermore, routing protocols are not only used to establish end-to-end routes but can also be applied to other research areas. For example, our research team is applying routing protocols for ad hoc networks to route construction for UAVs [35,36,37,38] and ORs may also be applicable for such environments when flexible route construction is required. Thus, the concept of ORs can be applied to any kind of graph to design routes, especially when dynamic topology and communication environment changes occur.

## 5. Conclusions

In this paper, we proposed a novel hierarchical OR protocol, VORTEX, to address the disadvantages and drawbacks of conventional routing protocols that put restrictions on network adaptability. In the proposed VORTEX, end terminals initiate their forwarding by simply broadcasting packets into a network and the terminal composing the network autonomously forwards them toward the desired destination terminal once the network has been set up. This allows endpoints to use the network as if it were a black box that can somehow deliver packets to desired destinations. Moreover, VORTEX performs the periodic tier update to adapt to dynamic topology changes in ad hoc networks. The simulation results show that VORTEX can achieve higher reliability with shorter delay as well as higher energetic efficiency. Therefore, we conclude that the proposed VORTEX realizes network-driven packet forwarding without using pre-established forwarding guides with reasonable network performance and also shows that the well-maintained hierarchical structure of VORTEX contributes to reusing or sharing routing information among the network for reducing routing cost.

Although VORTEX could achieve better performance than the conventional protocols from the above aspects, drawbacks such as an increase in the number of forwarders compared to conventional unicast routing protocols, which is essentially inevitable in ORs, still remain one of the issues to be addressed. However, suppressing unnecessary forwarding by using forwarding of other terminals as an implicit acknowledgment, which has been realized in another work [39], can deal with the drawbacks. Moreover, excluding infrequently used routed by measuring route usage can help suppress the number of forwarders. Furthermore, improving tier assignment or update procedure of VORTEX by dividing the hierarchy into smaller domains could be one of the solutions to the aforementioned limitation of VORTEX.

## Figures and Tables

**Table 1:** Routing protocols for multi-hop networks.

	Local Information-Based Routing		Network-Wide Information-Based Routing	
	Route-Based	Forwarder-Based	Route-Based	Forwarder-Based
Single path	DSR [2], FT-AORP [9]	AODV [3], CORA [10]	SOAR [11] ^1^, CEPRM [8]	GeRaF [12] ^1^
Multiple path	CHORUS [13] ^1^, CHOR [14] ^1^, EORB-TP [15] ^1^	VORTEX ^1^, AOMDV-GA [6]	HRAN [16], OxDSR [17] ^1^	ExOR [18,19] ^1^, LCAR [20] ^1^, TDiCOR [21] ^1^

^1^ Opportunistic Routing Protocol.

**Table 2:** HELLO message components.

*n*	Terminal ID
*d*	Degree of terminal
*t*	Generated time
*T*	Tier level of the terminal

## Data Availability

The data presented in this study are available on request from the corresponding author. The data are not publicly available due to NDA and licensing issue.

## References

[1] Clausen T.H., Jacquet P. (2003). Optimized Link State Routing Protocol (OLSR).

[2] Hu Y.C., Maltz D.A., Johnson D.B. (2007). The Dynamic Source Routing Protocol (DSR) for Mobile Ad Hoc Networks for IPv4.

[3] Das S.R., Perkins C.E., Belding-Royer E.M. (2003). Ad hoc On-Demand Distance Vector (AODV) Routing.

[4] Chakchouk N. (2015). A Survey on Opportunistic Routing in Wireless Communication Networks. IEEE Commun. Surv. Tutor..

[5] Liu H., Zhang B., Mouftah H.T., Shen X., Ma J. (2009). Opportunistic routing for wireless ad hoc and sensor networks: Present and future directions. IEEE Commun. Mag..

[6] Bhardwaj A., El-Ocla H. (2020). Multipath Routing Protocol Using Genetic Algorithm in Mobile Ad Hoc Networks. IEEE Access.

[7] Zhang H., Wang X., Memarmoshrefi P., Hogrefe D. (2017). A Survey of Ant Colony Optimization Based Routing Protocols for Mobile Ad Hoc Networks. IEEE Access.

[8] Quy V.K., Hung L.N., Han N.D. (2019). CEPRM: A Cloud-assisted Energy-Saving and Performance Improving Routing Mechanisms for MANETs. J. Commun..

[9] Hoang D.N.M., Rhee J.M., Park S.Y. (2022). Fault-Tolerant Ad Hoc On-Demand Routing Protocol for Mobile Ad Hoc Networks. IEEE Access.

[10] Abuashour A., Kadoch M. (2017). Performance Improvement of Cluster-Based Routing Protocol in VANET. IEEE Access.

[11] Rozner E., Seshadri J., Mehta Y., Qiu L. Simple opportunistic routing protocol for wireless mesh networks. Proceedings of the 2006 2nd IEEE Workshop on Wireless Mesh Networks.

[12] Zorzi M., Rao R. (2003). Geographic random forwarding (GeRaF) for ad hoc and sensor networks: Multihop performance. IEEE Trans. Mob. Comput..

[13] Yamamoto R., Ohzahata S., Kato T. A hierarchical opportunistic routing with stability information for mobile ad hoc networks. Proceedings of the 2016 International Conference on Advanced Technologies for Communications (ATC).

[14] Yamamoto R., Ohzahata S., Kato T. (2017). A Hierarchical Opportunistic Routing with Moderate Clustering for Ad Hoc Networks. IEICE Trans. Commun..

[15] Sang Q., Wu H., Xing L., Ma H., Xie P. (2020). An Energy-Efficient Opportunistic Routing Protocol Based on Trajectory Prediction for FANETs. IEEE Access.

[16] Trindade J., Vazão T. (2014). Routing on large scale mobile ad hoc networks using bloom filters. Ad Hoc Netw..

[17] Shen Q., Fang X., Kim S., He R. Combining opportunistic routing with dynamic source routing for wireless mesh networks. Proceedings of the IET International Communication Conference on Wireless Mobile and Computing (CCWMC 2009).

[18] Biswas S., Morris R. (2004). Opportunistic Routing in Multi-Hop Wireless Networks. SIGCOMM Comput. Commun. Rev..

[19] Biswas S., Morris R. (2005). ExOR: Opportunistic Multi-Hop Routing for Wireless Networks. Proceedings of the 2005 Conference on Applications, Technologies, Architectures, and Protocols for Computer Communications.

[20] Dubois-Ferrière H., Grossglauser M., Vetterli M. (2011). Valuable Detours: Least-Cost Anypath Routing. IEEE/ACM Trans. Netw..

[21] Kurth M., Zubow A., Redlich J.P. Cooperative Opportunistic Routing Using Transmit Diversity in Wireless Mesh Networks. Proceedings of the IEEE INFOCOM 2008—The 27th Conference on Computer Communications.

[22] Han M.K., Bhartia A., Qiu L., Rozner E. (2011). O3: Optimized Overlay-Based Opportunistic Routing. Proceedings of the Twelfth ACM International Symposium on Mobile Ad Hoc Networking and Computing.

[23] Hai L., Wang H., Wang J., Tang Z. (2014). HCOR: A high-throughput coding-aware opportunistic routing for inter-flow network coding in wireless mesh networks. EURASIP J. Wirel. Commun. Netw..

[24] Menon V.G., Prathap P.M.J. (2017). Towards Optimal Data Delivery in Highly Mobile Wireless Ad Hoc Networks. Int. J. Comput. Sci. Eng..

[25] Wang Z., Chen Y., Li C. (2012). CORMAN: A Novel Cooperative Opportunistic Routing Scheme in Mobile Ad Hoc Networks. IEEE J. Sel. Areas Commun..

[26] Zuo J., Dong C., Nguyen H.V., Ng S.X., Yang L.L., Hanzo L. (2014). Cross-Layer Aided Energy-Efficient Opportunistic Routing in Ad Hoc Networks. IEEE Trans. Commun..

[27] Wang Z., Chen Y., Li C. (2014). PSR: A Lightweight Proactive Source Routing Protocol For Mobile Ad Hoc Networks. IEEE Trans. Veh. Technol..

[28] Network Modeling. https://www.keysight.com/us/en/products/network-test/network-modeling.html.

[29] Chen Z., Zhou W., Wu S., Cheng L. (2020). An Adaptive on-Demand Multipath Routing Protocol With QoS Support for High-Speed MANET. IEEE Access.

[30] Okamura Y., Yamamoto R., Ohzahata S., Kato T. Opportunistic Routing for Heterogeneous IoT Networks. Proceedings of the 2019 IEEE International Conference on Consumer Electronics—Taiwan (ICCE-TW).

[31] Hosonuma E., Yamazaki T., Miyoshi T., Yamamoto R., Silverston T. (2021). On treating asymmetric links in backoff-based opportunistic routing: Problem and solution. IEICE Commun. Express.

[32] Kafaie S., Chen Y., Dobre O.A., Ahmed M.H. (2018). Joint Inter-Flow Network Coding and Opportunistic Routing in Multi-Hop Wireless Mesh Networks: A Comprehensive Survey. IEEE Commun. Surv. Tutor..

[33] Guan Q., Ji F., Liu Y., Yu H., Chen W. (2019). Distance-Vector-Based Opportunistic Routing for Underwater Acoustic Sensor Networks. IEEE Internet Things J..

[34] Ismail M., Islam M., Ahmad I., Khan F.A., Qazi A.B., Khan Z.H., Wadud Z., Al-Rakhami M. (2020). Reliable Path Selection and Opportunistic Routing Protocol for Underwater Wireless Sensor Networks. IEEE Access.

[35] Kokubun Y., Yamazaki T., Yamamoto R., Miyoshi T., Ueda K. (2022). Reactive route construction for UAV delivery considering travel time and safety using wireless multi-hop network. IEICE Commun. Express.

[36] Gunji H., Yamazaki T., Yamamoto R., Miyoshi T., Ueda K. (2022). Proactive route construction for UAV delivery considering distance and safety using wireless multi-hop network. IEICE Commun. Express.

[37] Gunji H., Yamazaki T., Yamamoto R., Miyoshi T., Ueda K. (2023). Methods for constructing collision avoidance route for multiple unmanned aerial vehicles using OLSR-based link hierarchization. IEICE Commun. Express.

[38] Kokubun Y., Yamazaki T., Yamamoto R., Miyoshi T., Ueda K. (2023). AODV-Based Routing Methods for UAVs Travel Time and Safety. IEICE Commun. Express.

[39] Yamazaki T., Yamamoto R., Miyoshi T., Asaka T., Tanaka Y. (2017). PRIOR: Prioritized Forwarding for Opportunistic Routing. IEICE Trans. Commun..

